# Evaluation of Hypoxia-Inducible Factor-1 Alpha (HIF-1α) in Equine Sarcoid: An Immunohistochemical and Biochemical Study

**DOI:** 10.3390/pathogens9010058

**Published:** 2020-01-14

**Authors:** Manuela Martano, Gennaro Altamura, Karen Power, Brunella Restucci, Francesca Carella, Giuseppe Borzacchiello, Paola Maiolino

**Affiliations:** 1 Department of Veterinary Medicine and Animal Productions, Naples University “Federico II”, Via F. Delpino 1, 80137 Naples, Italy; gennaro.altamura@unina.it (G.A.); karen.power@unina.it (K.P.); brunella.restucci@unina.it (B.R.); giuseppe.borzacchiello@unina.it (G.B.); paola.maiolino@unina.it (P.M.); 2 Department of Biology, University of Naples Federico II, MSA, 80126 Naples, Italy; francesca.carella@unina.it

**Keywords:** BPV, equine, HIF-1α, sarcoid, VEGF

## Abstract

Background: equine sarcoids are the most frequent skin tumors in equidae worldwide. It is well known that delta bovine papillomaviruses are their causative agents. We have recently shown the presence in equine sarcoids of abnormal vessel structures, which could cause a hypoxic condition. The aim of this study was to analyze the expression of hypoxia-inducible factor-1 alpha (HIF-1α) in a subset of BPV positive equine sarcoids and explore the relationship with vascular endothelial growth factor (VEGF) expression. Results: 80% of equine sarcoids showed strong cytoplasmic staining in >60% of neoplastic fibroblasts, while 20% of samples showed a moderate cytoplasmic staining in 40–60% of neoplastic fibroblasts for HIF-1α. Results of Western blotting (WB) were consistent with immunohistochemistry (IHC). Moreover, a positive correlation between HIF-1α and VEGF expression (r = 0.60, *p* < 0.01) was observed. Conclusion: we have shown that HIF-1α was strongly expressed in equine sarcoid. The upregulation of HIF-1α has been described in numerous tumors and can be modulated by many proteins encoded by transforming viruses. Thus, it is also possible that BPV could have a relevant role in HIF-1α pathway regulation, contributing to the development of equine sarcoids by promoting HIF-1α/VEGF mediated tumor angiogenesis.

## 1. Introduction

Equine sarcoids are the most frequent fibroblastic benign skin tumor in equidae worldwide, with prevalence rates of 0.5–2.0% [1,2]. They often form from scar tissue or sites of a previous wound or trauma, on the head, the limbs, the ventrum, and the paragenital region [3]. Sarcoids often persist, can be locally invasive, and rarely regress [3,4], although it has been recently reported that a high percentage of equine sarcoids spontaneously disappeared without therapy [5]. It is widely known that these lesions do not always respond to therapy and are often correlated with a high recurrence rate after surgical excision [3,4,6], leading to morbidity and impacting the function and aesthetics of affected equids, which decreases their economic value [7,8]. Equine sarcoids have a multifactorial cause [9]; however, it has been proved that there is a strong correlation between permanent infection with delta bovine papillomaviruses (BPV type 1, 2 and 13), and persistent and progressive sarcoids [7,10,11]. The evidence that BPV has a main role in the pathogenesis of sarcoids may be partly explained by the detection, in naturally occurring equine sarcoid, of BPV DNA in an episomal form and in multiple copies [11,12,13,14]. In addition, BPV oncogenes and capsid gene transcripts (E2, E5, E7, and L1) have been shown in equine sarcoid tissue [15,16,17], indicating the beginning of viral transcription and replication [12,18,19] and providing evidence for a direct involvement of BPV in equine sarcoids development. The protein E5, in synergy with E7, induces DNA damages and genomic instability [20,21,22,23], leading to the cell transformation and to subsequent development of cancer [12]. The principal mechanisms whereby the BPV major oncogene E5 induces malignant transformation, involve the activation of platelet-derived growth factor βeta-receptor (PDGFβ-r) [24], the immune evasion [11], the resistance to apoptosis [25], the upregulation of matrix metalloproteinase (MMPs) with consequent alteration of extracellular matrix (ECM) turnover [26], and the increase of angiogenesis [27]. Angiogenesis, the development of new vessels, is necessary for neoplastic invasion, growth, and metastasis, and has a crucial role in the development and progression of numerous human and animal cancers [28,29,30,31,32,33,34,35,36,37]. Angiogenesis is induced in response to hypoxia, and cellular response to hypoxia is primarily regulated through the activity of the hypoxia-inducible factor-1 (HIF-1) [38,39]. HIF-1 is a heterodimer composed by HIF-1alpha (HIF-1α) and HIF-1beta (HIF-1β) subunits. The latter, also known as aryl hydrocarbon receptor nuclear translocator, is constitutively expressed in the cell nucleus, whereas HIF-1α is synthesized continuously in an oxygen-dependent manner. Under normoxic conditions, HIF-1α is rapidly hydroxylated and degraded by prolyl hydroxylases (PHD). Under hypoxia, which is common in tumors, HIF-1α heterodimerizes with the HIF-1β subunit, and together, by translocating to the nucleus, they bind DNA and increase the transcription of target genes, such as vascular endothelial growth factor (VEGF) [40,41,42]. It is now accepted that VEGF expression is mediated by HIF-1α during hypoxia, and the expression of HIF-1α directly correlates with VEGF expression and tumor vascularity in several tumors [43,44,45,46,47,48,49,50,51]. Tumor hypoxia often appears in many cancers as a result of tumor growth exceeding its own angiogenic capability. In this regard, HIF-1α plays a crucial role in the adaptative response of neoplastic cells to oxygen limitation, inducing activation of numerous genes that are involved in angiogenesis. Moreover, besides being a ‘guardian’ of oxygen homeostasis, HIF-1α has recently emerged as a key player in energy metabolism, survival, cell migration, and in immune cell regulation [40,41,42,52,53].

We have recently shown an overexpression of VEGF and abnormal vessel structures [27] in equine sarcoids, which could cause a hypoxic condition, leading to an upregulation of HIF-1α.

Since the specific function of HIF-1α in sarcoid pathogenesis has not been investigated, so far, we analyzed the expression of HIF-1α in 35 BPV positive equine sarcoids and explored the relationship with VEGF expression reported in our previous study [27].

## 2. Results

### 2.1. Histological Features

The examined sarcoids (n = 35) showed the classic histological features of the lesion: epidermal hyperplasia with rete pegs; hyperkeratosis; proliferation of neoplastic fibroblast in the dermal layer, oriented in a ‘picket fence’ perpendicular to the basilar epidermal layer; exuberant extracellular matrix; presence of many small vessels irregular in shape [26,27].

### 2.2. Immunohistochemical Results

All results of HIF-1α expression pattern in 35 equine sarcoids and 10 normal skin samples are shown in Table 1.

#### 2.2.1. Control Samples

**Figure 1:**
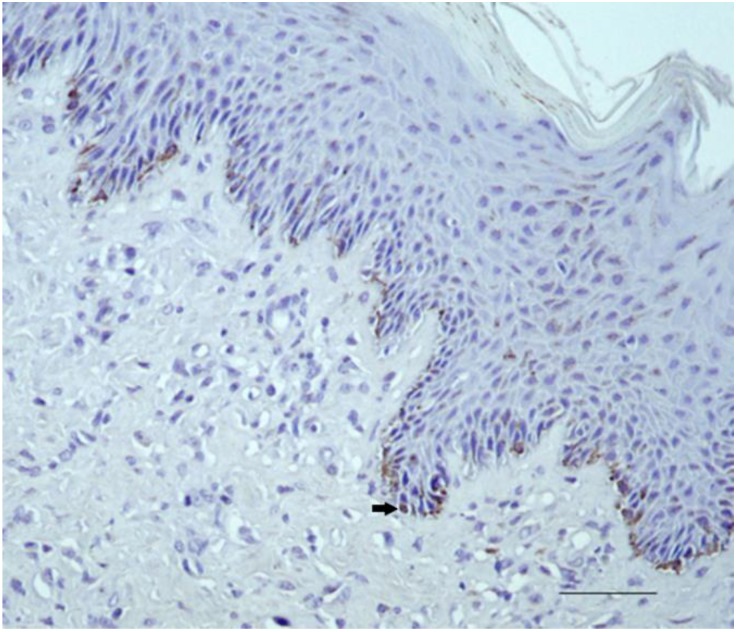
Equine normal skin. HIF-1α immunohistochemical staining. Expression in the basal epidermal cells (arrow) (40×). Scale bar: 100 μm.

Equine kidney used as positive control showed strong immunostaining for HIF-1α in tubular epithelial cells (Figure S1). Equine normal skin and sarcoid, used as the control, showed staining for Purified Rabbit IgG (P120–201-Bethyl Laboratories, Inc.) in cells at the base of the epidermis but not in fibroblasts (Figures S2 and S3). All 10/10 normal skin samples showed immunostaining for HIF-1α, in 10% of basal epidermal cells. Fibroblasts were negative (Figure 1).

#### 2.2.2. Sarcoid Samples

**Figure 2:**
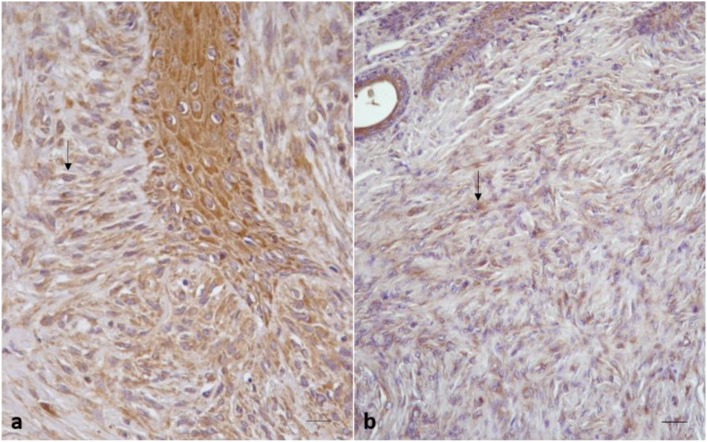
Equine sarcoid. HIF-1α immunohistochemical staining. (**a**) Strong immunohistochemical expression in sarcoid fibroblasts (arrow) (score 3; ++; 40×; Scale bar: 100 μm). (**b**) Moderate immunohistochemical expression in sarcoid fibroblasts (arrow) (score 2; +; 20×; Scale bar: 100 μm).

HIF-1α immunostaining was detected in all 35 equine sarcoid samples. In 28/35 sarcoid samples (80%), a strong immunolabeling was observed as finely granular cytoplasmic staining in >60% of neoplastic fibroblasts and endothelial cells (score 3; ++) (Figure 2a). Among these samples (score 3, ++), 100% were located on neck (2/2) and paragenital region (3/3), 90% (9/10) on limb, 83% (5/6) on head, 66% (4/6) were on abdomen, and 62% (5/8) on pectoral region.

The remaining 7/35 sarcoid samples (20%) showed a moderate granular cytoplasmic staining for HIF-1α in 40–60% of neoplastic fibroblasts (score 2, +) (Figure 2b). Among these samples (score 2, +), 10% (1/10) were located on limb, 34% (2/6) were located on abdomen, 38% (3/8) on pectoral region, and 17% (1/6) on head.

### 2.3. Statistical Results

The rank correlation analysis showed that the percentage of HIF-1α positive cells was positively correlated with the percentage of VEGF positive cells found during a previous study by our group (r = 0.60 *p* < 0.01) [27].

### 2.4. Biochemical Results

**Figure 3:**
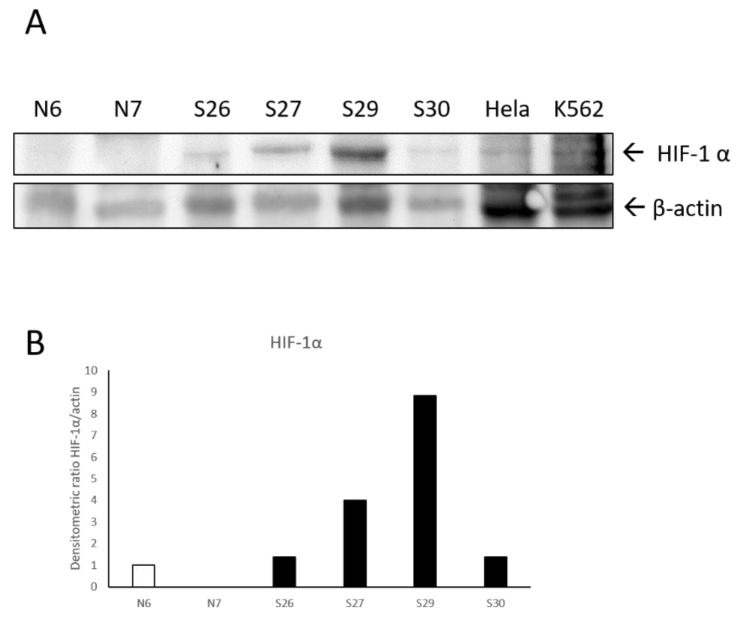
(**A**) Western blotting analysis showing overexpression of HIF-1α in equine sarcoids (S) compared to normal skin samples (N). Whole cell lysate from Hela cells and K562 cells was run concomitantly to ensure the specificity of the band. Blot was stripped and incubated with anti-β-actin antibody to perform normalization. (**B**) Densitometric analysis was performed with the results expressed as HIF-1α/actin ratio.

By Western blot, a band of the expected molecular size for HIF-1α (120 kDa, Figure S4) was identified in the tested samples, as well as in Hela and K562 cell lines used as positive control as suggested by antibody datasheet and literature data, confirming the specificity of the antibody (Figure 3A) [54]. HIF-1α was expressed at higher levels in sarcoid samples with respect to normal skin samples, where the band was detected at low to undetectable levels, as confirmed by densitometric analysis (Figure 3A,B).

## 3. Discussion

The insufficient levels of cellular oxygen, a condition also known as hypoxia, were demonstrated in many tumors [55] and were associated with a structural and functional abnormality of vessels or to an increase of oxygen consumption caused by the rapid proliferation of neoplastic cells [56]. HIF-1α has a relevant role in oxygen homeostasis, and experimental evidence has indicated that it is a major regulator of normal and tumor cell adaption to hypoxic stress [52,53,55,57]. HIF-1 is a heterodimeric protein composed of a constitutively expressed HIF-1β subunit and an O2-regulated HIF-1α subunit [58]. HIF-1α is degraded by the ubiquitin-proteasome pathway [59] under normoxic conditions, while it is protected from ubiquitination and proteasomal degradation under hypoxic-conditions [42]. After PHD inhibition, HIF-1α dimerizes in HIF-1β to form HIF-1, which is responsible for the transcription of genes encoding glucose transporters, glycolytic enzymes, and VEGF [40,41,42]. HIF-1α and VEGF are major regulators of angiogenesis [60] in the tumor microenvironment and have a crucial role in tumor progression [60,61,62]. As VEGF [27,29,32,34,60], HIF-1α is overexpressed in a large variety of tumors [60,63], and its association with unfavorable prognosis has been reported, as it activates genes that play a relevant role in angiogenesis, invasion, and metastasis [57,59,64].

In this study, we have observed, by immunohistochemistry and biochemical analysis, HIF-1α expression levels in BPV positive equine sarcoids, located in different body regions [27], and we have evaluated the correlation between HIF-1α and VEGF expression, previously analyzed in a study of ours.

In our samples, surprisingly, HIF-1α showed a cytoplasmic expression, while the antibody used by us (#ab114977, Abcam) was reported to have a nuclear expression. We hypothesize that the abnormal upregulation and accumulation of HIF-1α in the cytoplasm could be related to the inhibition of prolyl-hydroxylation (PHD) under hypoxia and to the consequent suppression of HIF-1α degradation, leading to its rapid accumulation in the cytoplasm [65]. HIF-1α shuttling between cytoplasm and nucleus is a complex process regulated by numerous factors [65], and it was already reported its cytoplasmic expression in a broad spectrum of tumors [66,67].

All normal skin samples showed negative immunostaining for HIF-1α in fibroblast, while a weak immunostaining was observed in the basal epidermal cells, where HIF-1α is known to be constitutively expressed. Moreover, 80% of sarcoid samples showed a strong and finely granular cytoplasmic staining for HIF-1α in >60% of sarcoid fibroblasts and endothelial cells, while in remaining samples (20%) the intensity of immunostaining was moderate and observed in 40–60% of neoplastic fibroblasts and endothelial cells.

Although the samples located on the neck, paragenital region, and limb showed higher intensity staining and percentage positive score (see Table 1), no correlation could be demonstrated.

In a previous study [27] including the same samples, we have reported VEGF overexpression in most keratinocytes, sarcoid fibroblasts, and endothelial cells. Moreover, we have recently shown that, even if small blood vessels were numerous, they showed irregularity in shape, and their lumina appeared indistinct. Taken together, these data strongly suggest that in sarcoid tissue, there could be a hypoxic condition in which HIF-1α would have a crucial role, leading to the upregulation of VEGF and an increase in the vessels’ number. However, despite the increase of vessels, they didn’t appear sufficiently mature, possibly inducing worse or persistent hypoxia, which in turn could induce the upregulation of HIF-1α and then of VEGF. This strict relationship between HIF-1α and VEGF was also, in part, evidenced in our statistical analysis results, which showed a positive correlation between HIF-1α and VEGF expression (r = 0.60; *p* < 0.01). Our results seem to suggest that HIF-1α not only could regulate VEGF expression but also contributes to the formation of a complex proangiogenic microenvironment in equine sarcoids, thereby affecting vessels’ morphology and, ultimately, the vessels’ function [68].

Numerous studies reported that HIF-1α synthesis, or its decreased degradation, can be modulated by different tumor suppressor genes and oncogenes, among which there are many proteins encoded by transforming viruses [58,69]. There is strong evidence that human papillomaviruses (HPV) oncoproteins can promote tumor angiogenesis via the upregulation of HIF1/VEGF pathways, specifically manipulating aspects of the cellular hypoxic response [70,71]. HPV E6 and E7 were shown to increase independently the induction of HIF-1α [65] or to interfere with HIF-1α degradation, leading to the inactivation of proteasomal degradation and to HIF-1α stabilization [39,72,73]. Moreover, built upon these evidences, and in light of the common biological functions of papillomavirus oncogenes [74], we may speculate that BPV oncoproteins could play a relevant role in regulation of HIF-1α pathway, contributing, at least in part, to the development of equine sarcoids by promoting HIF1α/VEGF mediated tumor angiogenesis.

Further investigations are needed to clarify the specific role of BPV in the regulation of HIF-1α/VEGF pathway, and to evaluate if there could be any correlation in equine sarcoid between HIF-1α and glucose transporter 1 (GLUT1), which is known to be the rate-limiting enzyme for glycolysis [75]. In hypoxic conditions, neoplastic cells have been reported to increase GLUT1 expression under the positive regulation of HIF-1α, leading to increase cellular glucose uptake, and support the aerobic glycolysis of cancer cells [75].

We believe that new knowledge of equine sarcoid pathogenesis would be necessary in order to gain new insights into the development of novel therapies for this pathology.

## 4. Materials and Methods

### 4.1. Tumor Samples

We analyzed 10 normal skin samples and 35 equine sarcoids. The normal skin samples, located on head (n = 3), abdomen (n = 2), neck (n = 2), limb (n = 2), and pectoral regions (n = 1), were obtained during necropsy from healthy horses. The equine sarcoids, located on the limbs (n = 10), pectoral region (n = 8), head (n = 6), abdomen (n = 6), paragenital (n = 3) and neck (n = 2) (Table 1), were surgically excised from affected horses, using best practice of veterinary care, according to Directive 2010/63/EU (art. 1), and processed for routine diagnosis and treatment. Each owner consented to use tissues for research purposes, according to the ethical guidelines of the Anatomic Pathology Diagnostic Service of the Department of Veterinary Medicine and Animal Production (University of Naples Federico II). All samples were 10% formalin-fixed, paraffin-embedded for routine histological processing, and stained with hematoxylin and eosin (HE). Four sarcoids (S26, S27, S28, S29) and 2 normal skin samples (N6–N7) were immediately frozen at −80 °C and analyzed by Western blotting. Al sarcoid samples, the same as those previously used [27], were BPV positive, while normal skin samples were BPV negative [24]. Moreover, no previous treatments with topical or intra-tumoral therapy prior to excision were used.

### 4.2. Immunohistochemistry

Sections (5 μm) were processed for immunohistochemistry using the streptavidin-biotin-peroxidase method. All sections were deparaffinized in alcohol decreasing solutions, and endogenous peroxidase activity was blocked by incubation in 0.3% H_2_O_2_ in methanol for 20 min. Antigen retrieval was performed by pre-treating with microwave heating (twice for 5 min each at 750 W) in citrate buffer, pH 6.0. The slides were washed three times with phosphate buffered saline (PBS, pH 7.4, 0.01 M), then incubated for 1 h at room temperature with normal goat serum (Santa Cruz Biotechnology, Santa Cruz, CA, USA) diluted at 20% in PBS. As primary antibody, a polyclonal rabbit anti-human (predicted to cross-react with horse) to anti-HIF-1α (#ab114977, Abcam; 775–826 amino acids at C terminal) diluted 1:100 in PBS was used and applied overnight at 4 °C. Control sections (equine normal skin and sarcoid) were incubated with PBS instead of the primary antibody and with rabbit IgG (Purified Rabbit IgG P120-201-Bethyl Laboratories, Inc.) at the same concentration as the primary antibody. Equine kidney sections were used as positive control and incubated with anti-HIF-1α antibody (#ab114977, Abcam; 775–826 amino acids at C terminal).

Then, sections were incubated with MACH 1 probe (Biocare Medical, LLC, Concord, CA, USA) for 20 min at room temperature and with MACH-1 Universal HPR-Polymer (Biocare Medical, LLC, Concord, CA, USA) for 30 min at room temperature. Sections were counterstained with hematoxylin, and the immunolabelling was revealed with diaminobenzidine tetrahydrochloride.

### 4.3. Scoring of Immunoreactivity

For the evaluation of HIF-1α expression, a semiquantitative score was applied by two independent observers (Martano M. and Maiolino P.) under blinded conditions. Briefly, for each sample, we have established the number of immunolabeled cells by counting 1000 cells in 10 fields at 400× magnification (40× objective 10× ocular), and we have expressed results as percentage and scored as follows: 0 (≤10% positive cells); 1 (10–40% positive cells); 2 (40–60% positive cells); 3 (>60% positive cells). Moreover, the intensity of immunostaining was graded, as performed in a previous study [27]: n.a. (not assessable), − (negative staining), +/− (weak immunostaining), + (moderate immunostaining), and ++ (strong immunostaining) (Table 1).

### 4.4. Statistical Analysis

Pearson correlation test was used to correlate the percentage of HIF-1α positive cells with the percentage of VEGF positive cells reported in a previous study [27].

### 4.5. Protein Extraction and SDS PAGE/Western Blotting

Tissue samples homogenization, protein extraction, denaturing polyacrylamide gel electrophoresis (SDS-PAGE), and WB was performed as previously described [27]. The membranes were subjected to blocking by using Tris buffered saline (TBS: 10 mM Tris-HCl, pH 7.4, 165 mM NaCl) added with 0.1% Tween 20 (TTBS) and 5% non-fat dry milk, at room temperature for 1 h. The anti-HIF antibody (#ab114977, Abcam) at 1:1000 dilution was incubated overnight at 4 °C.

Following four washing steps of 10 min in TTBS, donkey anti-rabbit secondary antibody conjugated with peroxidase was employed at 1:2000 dilution for 1 h at room temperature. After additional washing steps, bound antibody was visualized by enzyme chemiluminescence with Clarity ™ Western ECL Blotting Substrate (Bio-Rad Laboratories, Milano, Italy). The blots were stripped and reprobed for β-actin (CP01, Calbiochem, San Diego, CA, USA) (1:500) as a loading control in order to perform normalization. Protein quantization and normalization were performed as reported in a previous study [27].

## 5. Conclusions

Finally, in our study, we have demonstrated for the first time the increase of HIF-1α expression in equine sarcoid, and we hypothesized that HIF-1α, together with VEGF, could have a role in sarcoid development.

Recent advances in cancer biology at the cellular and molecular levels highlighted the HIF-1α pathway as a crucial survival pathway for which novel strategies of cancer therapy could be developed [76]. We hope that in the future, hypoxia-inducible factor-1 (HIF-1) could be an important cancer drug target for equine sarcoid, since not all sarcoids are responsive to therapy, despite the numerous treatment choices available.

## Figures and Tables

**Table 1:** Immunoreactivity scoring of HIF-1α in 35 equine sarcoids.

Location	Number of Cases	Staining Intensity Score *	Percentage Positive Score **
Neck	2	++	3
Limb	9	++	3
	1	+	2
Abdomen	4	++	3
	2	+	2
Pectoral region	5	++	3
	3	+	2
Head	5	++	3
	1	+	2
Paragenital	3	++	3

* Staining intensity score: + moderate immunolabelling, and ++ strong immunolabelling. ** Percentage positive score: 0 (≤10% positive cells), 1 (10–40% positive cells), 2 (40–60% positive cells), and 3 (>60% positive cells).

## Supplementary Materials

The following are available online at https://www.mdpi.com/2076-0817/9/1/58/s1. Figure S1: Equine kidney used as positive control showed strong immunostaining for HIF-1α in tubular epithelial cells; Figure S2: Equine normal skin showed staining for Purified Rabbit IgG (P120-201-Bethyl laboratories-INC) in cells at the base of the epidermis but not in fibroblasts; Figure S3: Equine sarcoid showed staining for Purified Rabbit IgG (P120-201-Bethyl laboratories-INC) in cells at the base of the epidermis but not in fibroblasts; Figure S4: Full length membrane of Western blotting for HIF-1 α from Figure 3, demonstrating detection of a band at the expected molecular size (~120 kDa) in equine tissue samples, Hela and K562 cell lines. Molecular markers (M) are shown, molecular weights in kDa are indicated on the left of each M band.

The data sets supporting the results of this article are included within the article and its additional files.

## Abbreviations

BPV-1	Bovine papillomavirus type-1
BPV-2	Bovine papillomavirus type-2
BPV-13	Bovine papillomavirus type-13
HPVs	Human Papillomaviruses
HIF1	Hypoxia-Inducible Factor-1 alpha (HIF-1α)
VEGF	Vascular Endothelial Growth Factor
IHC	Immunohistochemistry
WB	Western Blotting
N	normal skin
PDGFβ-r	Platelet-Derived Growth Factor βeta-receptor
MMPs	Matrix Metalloproteinase
ECM	Extracellular Matrix
PHD	Prolyl Hydroxylases
