# Supplementary figures and images for: Evaluation of Hypoxia-Inducible Factor-1 Alpha (HIF-1α) in Equine Sarcoid: An Immunohistochemical and Biochemical Study
Source: Pathogens. 2020 Jan 14;9(1):58. doi: 10.3390/pathogens9010058 (PMC7168668; doi:10.3390/pathogens9010058)

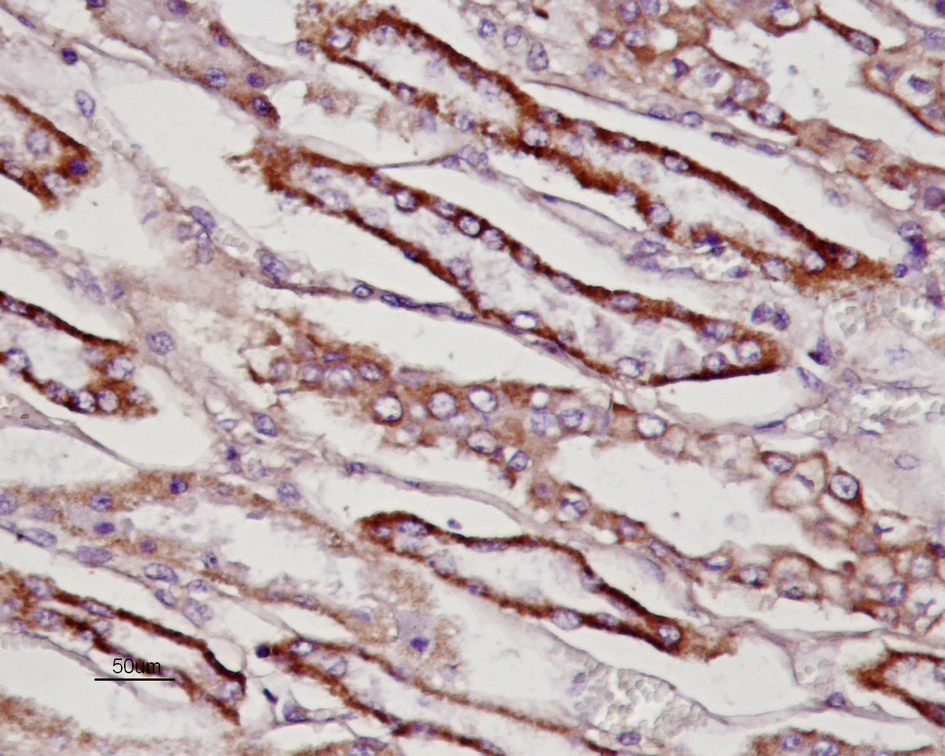

Supplement: Supplementary file 1 [file pathogens-09-00058-s001.zip › S1.tif]

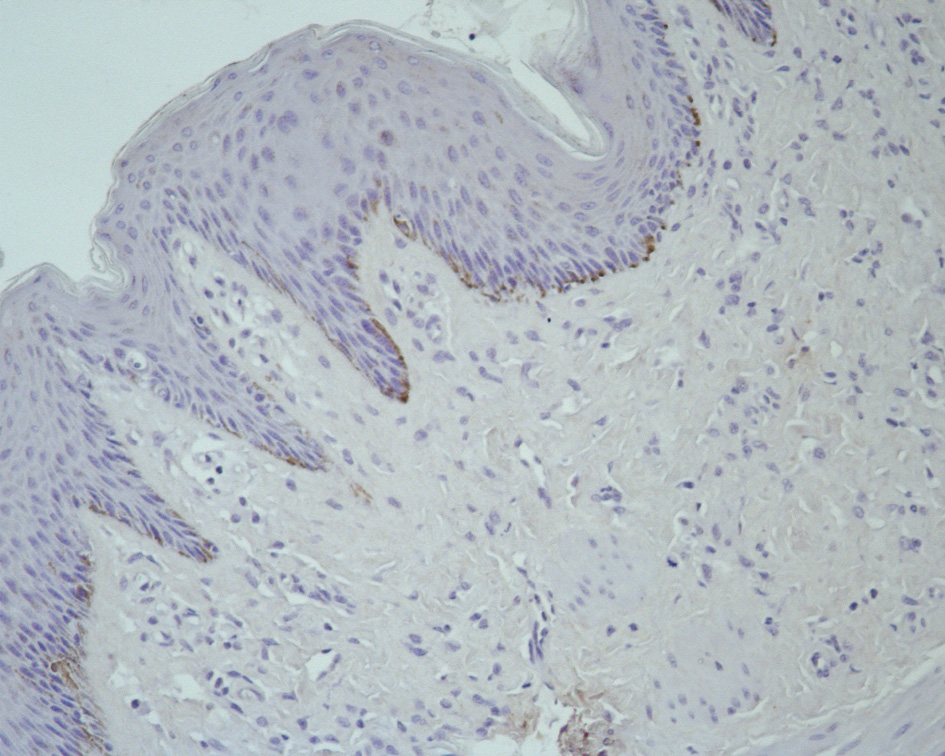

Supplement: Supplementary file 1 [file pathogens-09-00058-s001.zip › S2.tif]

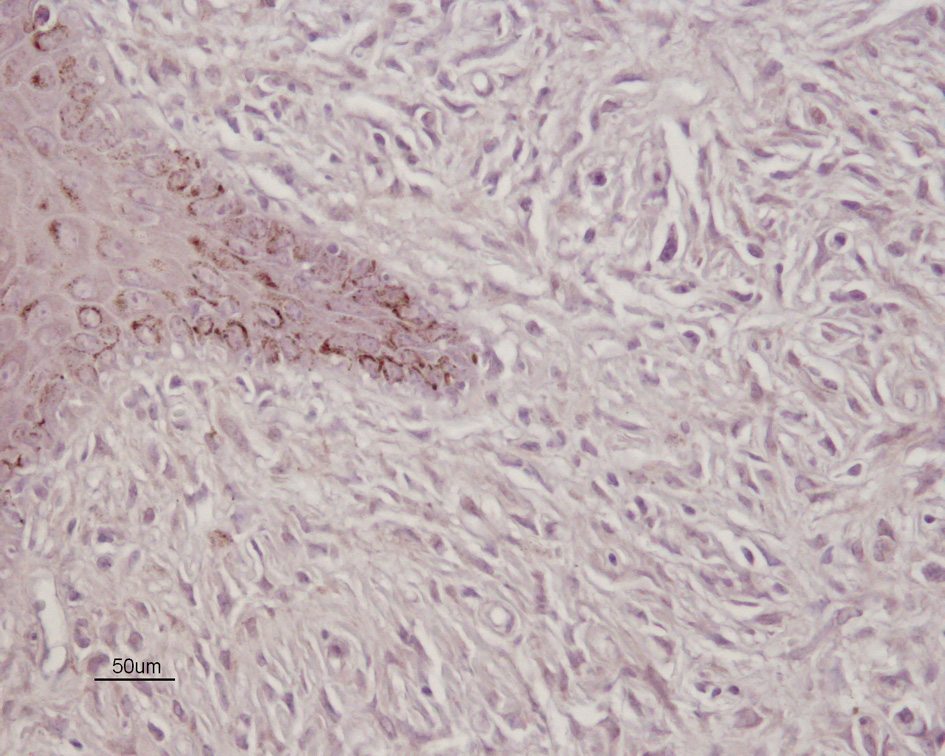

Supplement: Supplementary file 1 [file pathogens-09-00058-s001.zip › S3.tif]

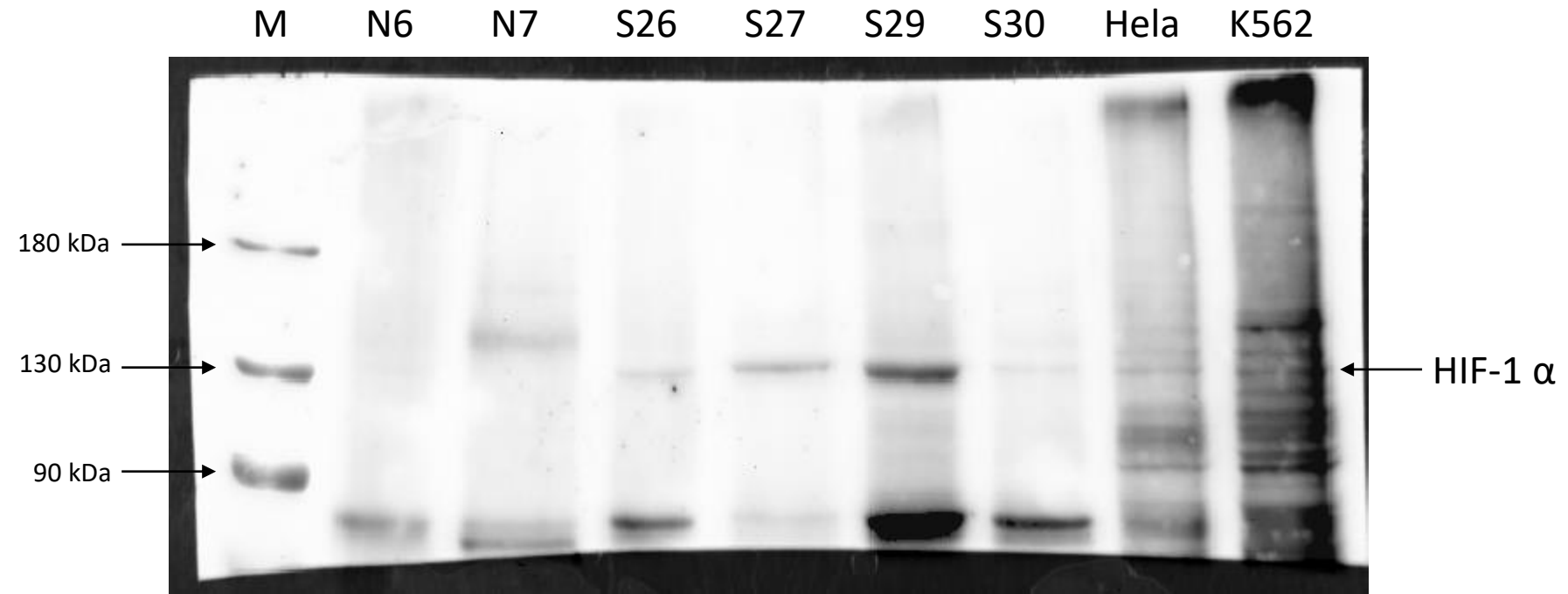

Supplement: Supplementary file 1 [file pathogens-09-00058-s001.zip › S4.pdf]
